# Enhanced Mechanical Stability of Water-Based Peel-Off Nail Polish Through Riboflavin Phosphate-Mediated Visible Light Photocrosslinking

**DOI:** 10.3390/polym17060766

**Published:** 2025-03-14

**Authors:** Minjin Kim, Youngran Park, Hyun Jong Lee

**Affiliations:** 1 School of Chemical, Biological and Battery Engineering, Gachon University, 1342 Seongnam-daero, Seongnam-si 13120, Republic of Korea; alswls8848@naver.com; 2 Pluchke Co., Ltd., 20 Dongtancheomdansaneop 1-ro, Hwaseong-si 18469, Republic of Korea; yrpark79@pluchke.com

**Keywords:** visible-light-induced photocrosslinking, riboflavin phosphate, water-based polyurethane, peel-off nail polish, dual-network structure

## Abstract

Water-based peel-off nail polishes offer environmental and safety advantages but often suffer from poor mechanical properties. This study investigated the effect of visible-light-induced photocrosslinking with riboflavin phosphate (RFP) on the mechanical properties and adhesion of water-based peel-off nail polish films. Polyurethane films that contained various concentrations of RFP (0–0.1%) were prepared by combining two commercial water-based polyurethane dispersions and characterized through tensile testing, rheological analysis, and adhesion measurements. Under large deformation, the photocrosslinked films showed significantly enhanced mechanical properties, with the highest RFP concentration (0.1%) exhibiting a 54% increase in tensile strength and a 94% increase in Young’s modulus compared with the control, which reflected a transition from physical to covalent network dominance. Rheological analysis under small deformation revealed the formation of complex network structures, where lower RFP concentrations maintained a higher chain mobility beneficial for adhesion, while higher concentrations created more stable networks with enhanced thermal stability, which maintained 50% of the initial storage modulus up to 100 °C. The films exhibited biocompatibility across all RFP concentrations in the cell viability tests, and the straightforward preparation process that used commercially available materials suggests immediate potential for industrial implementation. These results demonstrate that RFP-mediated visible light photocrosslinking offers a promising approach for developing high-performance, environmentally friendly nail polish formulations that combine enhanced durability with user safety and manufacturing practicality.

## 1. Introduction

Nail polishes are crucial products in the cosmetic industry, where they are widely used to enhance the appearance and durability of nails. Beyond their aesthetic appeal, nail polishes serve as an important protective function by reinforcing fragile nail surfaces against daily wear and environmental damage [1,2]. Traditional nail polishes face several critical challenges. These formulations typically consist of various solid components dissolved in organic solvents, forming a clear or pigmented film upon application and drying. However, they raise significant environmental and health concerns due to their high volatile organic compound (VOC) content [3]. The reliance on organic solvents not only contributes to environmental pollution but also poses potential health risks through prolonged exposure during application and wear [4].

Gel nail polishes emerged as an advanced solution that is capable of forming cross-linked networks under ultraviolet (UV) radiation, thereby offering enhanced durability and improved mechanical properties. These formulations are generally applied in three layers: a basecoat, a colored polish layer, and a clear topcoat, each cured with UV radiation [1]. While this multi-layer system combined with crosslinking processes contributes to improved longevity and resistance to chipping, the use of UV nail dryers has raised significant health and safety concerns due to the potential risks of skin cancer associated with prolonged UV exposure [5,6,7]. Additionally, the removal process typically requires aggressive organic solvents or mechanical scraping, which can damage the natural nail surface.

Water-based peel-off nail polishes represent another promising direction, offering numerous advantages, including minimal VOC emissions, easy removal without harsh chemicals, and a reduced risk of nail damage [8]. These formulations typically use water-dispersed polymers that form a film on the nail surface, which can be easily peeled off without chemical solvents [9]. This characteristic makes them particularly appealing for consumers concerned about potential damage caused by traditional nail polish removers. However, a major limitation of existing water-based peel-off nail polishes is their low mechanical strength, which significantly impacts their durability and overall performance [9].

Water-based polyurethane dispersions present promising base materials for peel-off nail polishes due to their excellent flexibility, film-forming ability, and compatibility with various additives [10,11,12,13]. Through a strategic combination of different polyurethane dispersions, it is possible to create balanced systems that leverage their complementary properties. For example, high-molecular-weight dispersions can provide enhanced mechanical strength and durability, while lower-molecular-weight variants contribute to improved film formation and flexibility [14]. However, the mechanical strength of these polyurethane films alone may be insufficient for high-performance applications, particularly in maintaining durability under daily wear conditions [15].

Riboflavin phosphate (RFP), a derivative of vitamin B2, offers an innovative solution as a photoinitiator for visible-light-induced photocrosslinking [16,17,18]. Its unique ability to absorb visible light and initiate crosslinking reactions in polymer matrices makes it an ideal candidate for safer and more user-friendly photocuring processes [17,18]. When exposed to visible light, RFP generates reactive species that facilitate the formation of cross-linked networks, enhancing the mechanical properties without compromising adhesion [16]. Furthermore, as a naturally occurring vitamin derivative, RFP presents a more biocompatible option compared with synthetic photoinitiators.

This study aimed to develop a novel water-based peel-off nail polish system that overcomes the limitations of existing formulations through several innovative approaches. First, we utilized visible-light-responsive RFP as a photoinitiator, eliminating UV exposure risks while maintaining efficient curing. The longer wavelength of visible light (400–500 nm) compared with UV radiation (typically 365 nm) results in lower energy exposure to the surrounding tissues, potentially reducing health concerns associated with radiation exposure. Second, we strategically combined commercial water-based polyurethanes to create an optimized base matrix with balanced properties that, once dried, forms a water-resistant film that maintains durability during normal use while allowing for easy peel-off removal without solvents or mechanical abrasion that could damage the natural nail.

By systematically investigating the effect of RFP-mediated visible light photocrosslinking on mechanical properties and adhesion, we aimed to establish a new paradigm in nail polish formulation. This research addressed not only the immediate concerns of mechanical durability and ease of removal but also broader issues of environmental sustainability and user safety. The potential outcomes could lead to the development of a new generation of nail polish products that combine the ease of removal characteristic of peel-off formulations with the durability typically associated with gel polishes, all while using a safer, visible light curing process.

## 2. Materials and Methods

### 2.1. Materials

Unless otherwise stated, all chemicals and solvents were used as provided by the manufacturer. Commercial water-based polyurethane dispersions SUD960 and Akuarane5015 were obtained from Terrachem (Gyeonggi, Republic of Korea) and T&L (Gyeonggi, Republic of Korea), respectively. Riboflavin phosphate (RFP), used as a photoinitiator, was purchased from Sigma-Aldrich (Milwaukee, WI, USA). Ethanol and distilled water were used as solvents and were purchased from Duksan Pure Chemicals (Gyeonggi, Republic of Korea). NIH/3T3 fibroblast cells, used for the cytotoxicity evaluation, were purchased from the American Type Culture Collection (ATCC, Manassas, VA, USA). Cell culture media and reagents, including Dulbecco’s Modified Eagle Medium (DMEM), fetal bovine serum (FBS), penicillin/streptomycin (P/S), and trypsin/EDTA, were purchased from Gibco (Waltham, MA, USA). The MTT reagent for cell viability assays was purchased from Thermo Fisher Scientific (Waltham, MA, USA).

### 2.2. Synthesis and Fabrication of Photocrosslinkable Films

Different concentrations of riboflavin phosphate (0%, 0.025%, 0.05%, 0.075%, 0.1%) were added to the polymer solution to investigate the effects of RFP on the film properties. The polymer solution was prepared by mixing SUD960 (viscosity: 500 cps) and Akuarane5015 (viscosity: 100 cps) in a 1:1 ratio, which was determined to be optimal based on preliminary studies that balanced the mechanical strength and elongation properties essential for nail polish applications (Figure S1). The mixture was stirred thoroughly using a magnetic stirrer for 30 min to ensure an even distribution of the photoinitiator.

The prepared polymer solution was cast onto clean glass plates using a doctor blade with a gap setting of 1 mm to achieve a uniform thickness. The films were then placed in a convection oven at 37 °C for 24 h to facilitate solvent evaporation. At the onset of the drying process, visible light curing was performed using a 1W LED curing light (wavelength range: 400–500 nm). The light source was positioned 1 cm away from the sample surface, and the exposure time was 30 min. Following the light exposure, the films continued to dry in the convection oven for the remainder of the 24 h period. After the drying process was complete, the films were allowed to cool to room temperature.

### 2.3. Physicochemical Characterization Methods

The chemical characteristics of the films were analyzed using Fourier-transform infrared (FT-IR) spectroscopy (iS50, Thermo Fisher Scientific, Waltham, MA, USA). FT-IR spectra were recorded to investigate the chemical changes and crosslinking in the polymer films. The samples were scanned in the range of 4000–400 cm^−1^.

The mechanical properties of the films were evaluated using a 34sc-1 universal testing machine (UTM) instrument (Instron, Canton, MA, USA) at the Smart Materials Research Center for IoT, Gachon University, Republic of Korea. The tensile strength and Young’s modulus were measured by cutting the films into standard test strips of 1 cm × 5 cm and testing them at a crosshead speed of 5 mm/min. The elongation at break was also measured using the same UTM setup.

Rheological measurements were performed using a stress-controlled rheometer (MCR92, Anton Paar, Graz, Austria) equipped with a parallel plate geometry (diameter: 25 mm). Samples were prepared as circular discs with a diameter of 25 mm by cutting from the fully formed and light-exposed films after the complete drying process. Prior to the measurements, a strain sweep test was conducted from 0.01% to 100% strain at 1 Hz to determine the linear viscoelastic region (LVR) for each sample.

Frequency sweep measurements were carried out within the identified LVR, typically at 0.5% strain, over a frequency range of 0.1 to 100 Hz at 25 °C. Temperature sweep tests were performed from 25 °C to 100 °C at a heating rate of 3 °C/min and a fixed frequency of 1 Hz. The storage modulus (G′), loss modulus (G″), and tan δ were recorded for all measurements.

Adhesion properties were assessed through lap shear tests to evaluate the adhesion of the films to the polyethylene terephthalate (PET) substrate. For the adhesion testing, approximately 100 μL of polymer solution was applied between two PET films over an area of 2 cm × 2 cm. The visible light curing was performed using a 1W Blue Light Plate positioned 1 cm away from the sample surface for 30 min, followed by drying in a convection oven at 37 °C for 24 h. Lap shear tests were conducted using a UTM instrument. The ends of the PET films were secured in the testing jigs, and the samples were pulled at a crosshead speed of 5 mm/min. A pre-test at 2 mm/min was conducted to ensure a proper alignment and contact. These tests provided data crucial for determining the suitability of the films for nail polish applications.

### 2.4. In Vitro Biocompatibility Assessment

The cytotoxicity of the polymer films was assessed using the MTT assay to determine the cell viability. Extracts from the films were prepared by soaking them in PBS at 37 °C for 24 h. These extracts were then added to the cell culture medium at a 10% concentration. NIH/3T3 fibroblast cells were incubated with the extract-containing medium for 24 h. After the incubation, the cell viability was assessed by adding the MTT reagent and allowing it to react for 2 h. The formazan crystals were then dissolved in DMSO, and the absorbance was measured at 540 nm using a microplate reader (BioTek, Winooski, VT, USA).

### 2.5. Statistical Analysis

Statistical analysis was conducted to determine the significance of the results obtained from the various tests. Data are expressed as the mean ± standard deviation. Comparisons between groups were made using one-way analysis of variance (ANOVA), followed by Tukey’s post hoc test where appropriate, using GraphPad Prism 10 software. A *p*-value of less than 0.05 was considered statistically significant. Statistical significance is denoted as follows: * *p* < 0.05 and ** *p* < 0.01.

## 3. Results and Discussion

### 3.1. Mechanism and Characterization of RFP-Mediated Photocrosslinking

Riboflavin phosphate (RFP), a water-soluble derivative of vitamin B2, serves as an effective visible-light-responsive photoinitiator. RFP absorbs light at wavelengths of 380 and 450 nm and forms reactive singlet oxygen in the presence of oxygen [16]. This photochemical property enables RFP to initiate crosslinking reactions under safer visible light conditions compared with traditional UV-based systems, making it particularly attractive for biomedical applications [19,20,21,22].

To develop an effective polyurethane film for nail polish applications, we strategically combined two different water-based polyurethane dispersions, SUD960 and Akuarane5015, in a 1:1 ratio. This combination was crucial in creating a base film with suitable properties before introducing the photocrosslinking effect of the RFP.

SUD960 and Akuarane5015, both with solid contents of 30–35%, are commercially available water-based polyurethane dispersions that exhibit significantly different viscosities (500 cps and 100 cps, respectively), indicating a difference in their molecular weights. These materials are particularly suitable for enhancing water-based polyurethane films, as they can be readily integrated into existing commercial products due to their established manufacturing processes.

The contrasting properties of these individual polymers guided our formulation strategy. SUD960 forms films with high strength and high elasticity but low elongation, resulting in a rigid nature. In contrast, Akuarane5015 produces films with lower strength and elasticity, but higher elongation, leading to a more flexible character. By combining these polymers in equal proportions, we aimed to compensate for their individual limitations. However, this combination strategy, while balancing the overall properties, resulted in a significant decrease in mechanical strength—a critical drawback for practical applications. To address this limitation, we introduced RFP-mediated photocrosslinking as an additional strengthening mechanism.

**Figure 1:**
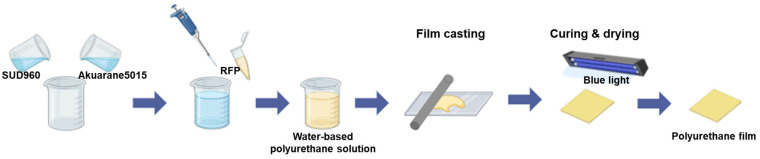
Schematic illustration of the fabrication process of water-based polyurethane films incorporating RFP.

**Figure 2:**
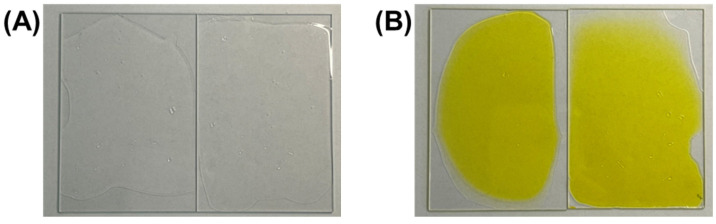
Optical appearance of water-based polyurethane films with and without RFP photocrosslinking: (**A**) transparent control film without RFP (R0), demonstrating the inherent optical clarity of the polyurethane matrix, and (**B**) the film containing 0.1% RFP (R0.1) exhibiting the characteristic yellow tint due to riboflavin incorporation while maintaining a uniform film formation and structural integrity.

Figure 1 illustrates the fabrication process of the RFP-containing polyurethane films. The successful formation of uniform films is evident in Figure 2, which compares the visual appearance of films without RFP (R0) and with 0.1% RFP (R0.1). The R0 film exhibited transparency, which is characteristic of well-formed polyurethane films. The R0.1 film displayed a slight yellow tint due to the presence of RFP, while it maintained the film’s overall morphology and appearance, indicating that the RFP integration did not disrupt the film-forming capabilities of the polyurethane blend.

**Figure 3:**
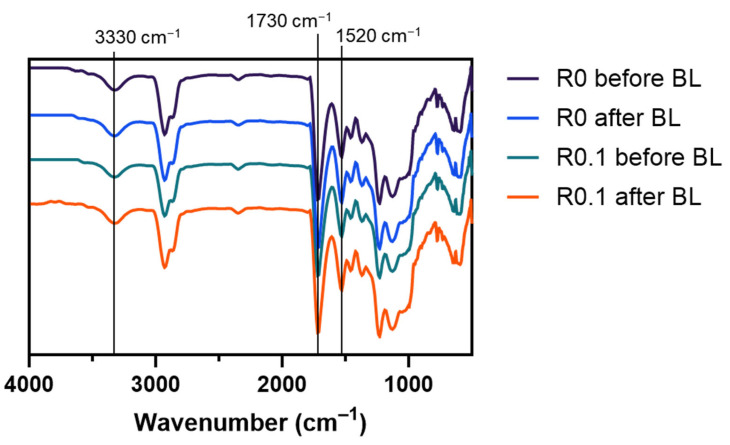
FT-IR spectra of water-based polyurethane films: control film (R0) and RFP-containing film (R0.1) before and after blue light (BL) exposure.

To investigate the chemical composition of the films, we conducted a Fourier-transform infrared (FT-IR) spectroscopy analysis (Figure 3). The FT-IR spectra revealed characteristic peaks associated with polyurethane: N-H stretching (~3330 cm^−1^), C=O stretching (~1730 cm^−1^), and C-N stretching (~1520 cm^−1^). The spectra remained consistent across all samples from the pristine film (R0) to the RFP-incorporated films after the light exposure.

This spectral consistency indicates that the incorporation of RFP and subsequent photocrosslinking process preserved the primary chemical structure of the polyurethane matrix. Any chemical modifications that resulted from the photocrosslinking reaction occurred at concentrations below the detection limit of the FT-IR spectroscopy, given that RFP was present in relatively small quantities compared with the bulk polymer matrix. The maintenance of the original chemical structure suggests favorable chemical stability of the formulation, which is essential for cosmetic applications.

The preservation of the chemical structure while achieving enhanced mechanical properties through RFP-mediated photocrosslinking demonstrated the advantages of our approach. This strategy enabled the reinforcement of polyurethane films without compromising their inherent chemical properties, which is particularly crucial for applications that require both mechanical durability and chemical stability. The combination of commercially available polyurethanes with RFP-mediated photocrosslinking provides a practical pathway for developing improved water-based nail polish formulations that can be readily implemented in current manufacturing processes.

The timing of the light exposure in relation to the drying process is an important consideration in this system. In our protocol, visible light curing was performed at the onset of drying when the polymer matrix contained sufficient moisture to facilitate the RFP mobility and reactivity. The relationship between the moisture content, crosslinking efficiency, and potential effects of ambient light exposure after the film formation represents an interesting area for future investigation to further optimize the crosslinking mechanism in these water-based systems.

### 3.2. Structure–Mechanical Property Relationships of Photocrosslinked Networks

**Figure 4:**
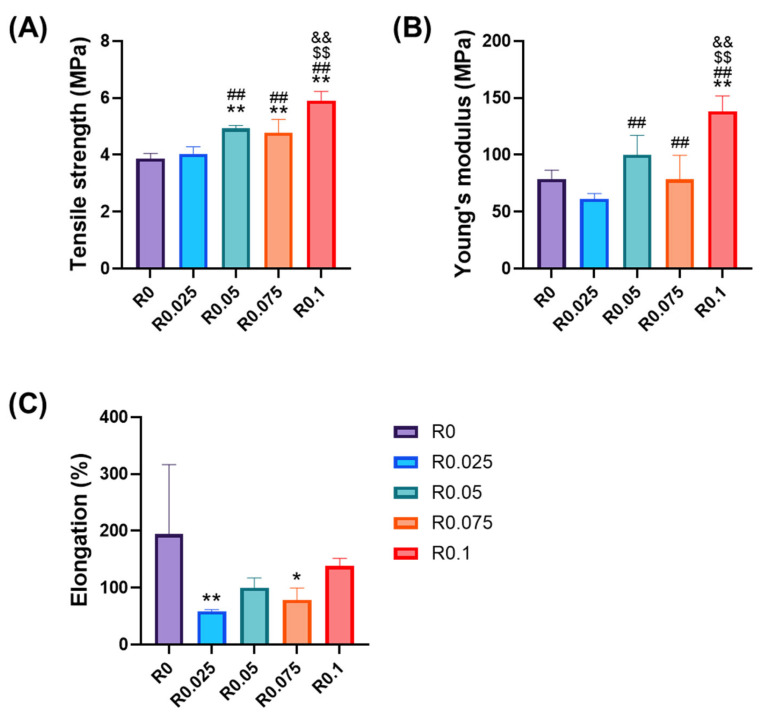
Mechanical properties of polyurethane films with various RFP concentrations (R0–R0.1): (**A**) tensile strength, (**B**) Young’s modulus, and (**C**) elongation at break. Statistical significance compared with R0 (* *p* < 0.05, ** *p* < 0.01), R0.025 (## *p* < 0.01), R0.05 ($$ *p* < 0.01), and R0.075 (&& *p* < 0.01) (n = 5).

The mechanical properties of photocrosslinked films were systematically evaluated through tensile testing to understand the complex relationship between the RFP concentration and network formation (Figure 4). The incorporation of the RFP and subsequent visible light exposure significantly influenced the mechanical characteristics of the films, which revealed a non-linear concentration-dependent behavior that provided insight into the underlying network formation mechanisms.

Tensile strength exhibited an initial increase with the RFP concentration from 3.88 ± 0.15 MPa for the control (R0) to 4.93 ± 0.09 MPa at a moderate RFP level (R0.05) (Figure 4A). This enhancement can be attributed to the formation of an optimal network structure where photoinitiated crosslinks effectively reinforced the polymer matrix without significantly disrupting the inherent polymer chain organization [23]. While R0.075 showed a slight decrease in the tensile strength, this reduction was not statistically significant. The overall trend demonstrated a progressive increase in the tensile strength with increasing RFP concentration, ultimately reaching 5.90 ± 0.30 MPa for R0.1, representing a significant enhancement in the mechanical performance.

Young’s modulus demonstrated a similar non-linear trend with increasing RFP concentration (Figure 4B). The control film (R0) exhibited a modulus of 78.89 ± 6.95 MPa, which initially increased to 100.09 ± 15.23 MPa at R0.05. This intermediate concentration appears to represent a critical point in the network formation, beyond which the relationship between the crosslinking density and mechanical properties became more complex. The highest RFP concentration (R0.1) ultimately achieved 138.28 ± 12.00 MPa, representing a 75% improvement over the control. Although slight decreases were observed at R0.025 and R0.075, these reductions were not statistically significant, and the overall trend showed an increase in Young’s modulus with increasing RFP concentration.

The non-linear relationship between the RFP concentration and mechanical properties provides insights into the complex interplay between the chemical crosslinking and physical interactions in network formation. At a low RFP concentration (0.025%), the introduction of sparse covalent bonds through photocrosslinking appeared to disrupt the original molecular alignment of the polyurethane chains, which partially interfered with their physical interactions without providing sufficient crosslinking density to compensate, which resulted in the observed decrease in Young’s modulus compared with R0.05. The network continued to evolve as the RFP concentration increased, where the highest concentration (0.1%) ultimately achieved the most robust mechanical properties.

This transition from a physically dominated to a covalently dominated network structure led to not only an increased crosslinking density but also improved the crosslinking homogeneity throughout the polymer network. Recent studies demonstrated that crosslinking homogeneity plays a more critical role than the crosslinking density in determining the mechanical properties of polyurethanes [24]. At higher RFP concentrations (0.05–0.1%), the increased availability of photoinitiator molecules likely promoted a more uniform distribution of crosslinks, which resulted in a more homogeneous network structure. This enhanced network uniformity, rather than a merely increased crosslinking density, could be the primary factor that contributed to the observed improvements in the tensile strength and Young’s modulus. This interpretation aligns with recent findings that demonstrate the correlation between the crosslinking homogeneity and mechanical property enhancement in polyurethane networks [24].

The elongation behavior further supports this network evolution mechanism (Figure 4C). The control films (R0) showed substantial elongation at break (194 ± 109%), primarily governed by physical interactions between polymer chains. The initial decrease in elongation with RFP addition reflects the disruption of these physical interactions, while the relatively consistent elongation values among the higher RFP concentrations suggest the establishment of a stable, covalently crosslinked network structure that maintains certain molecular mobility.

These findings demonstrate that the mechanical properties were determined by the balance between covalent crosslinks and physical interactions, with the covalent bonds becoming the dominant factor at higher RFP concentrations. This understanding provides valuable guidance for optimizing the crosslinking density in photocrosslinked systems.

### 3.3. Dynamic Mechanical Behavior and Network Characteristics

**Figure 5:**
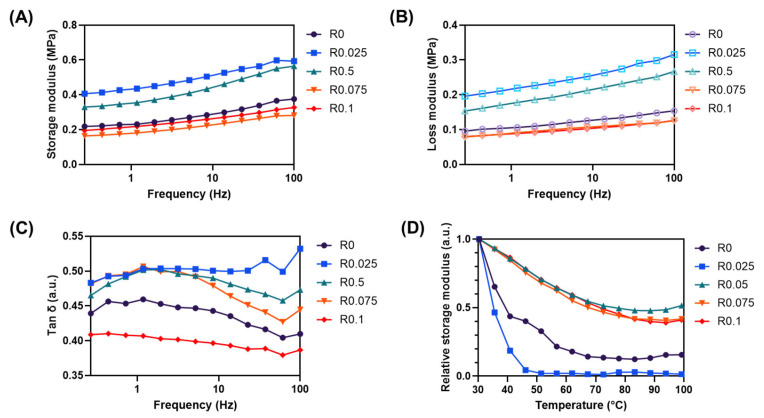
Rheological analysis of polyurethane films with various RFP concentrations (R0–R0.1): (**A**) storage modulus (G′), (**B**) loss modulus (G″), (**C**) tan δ as a function of frequency, and (**D**) temperature-dependent relative G′ measured at 1 Hz in the range of 25–100 °C.

The viscoelastic properties and network formation mechanisms of our photocrosslinked films were investigated through comprehensive rheological measurements under small deformation conditions, which provided complementary insights into the large deformation tensile testing results (Figure 5). Small deformation behavior is governed by linear viscoelastic theory, where both physical interactions and covalent bonds contribute to the material response, while large deformation involves nonlinear behavior where the disruption of physical interactions becomes more significant [25].

Frequency sweep measurements unveiled an intriguing trend in viscoelastic properties (Figure 5A,B). Films with low RFP concentrations (0.025% and 0.05%) exhibited notably higher storage (G′) and loss (G″) moduli compared with the control under small deformation conditions. This behavior, which appears to contradict the tensile test results, can be explained by the different deformation regimes involved in each measurement. Under a small deformation, both physical interactions and newly formed covalent bonds collectively contribute to the material response, resulting in higher moduli. However, under the large deformation conditions of tensile testing, the disruption of physical interactions by introduced covalent bonds becomes more pronounced, leading to reduced mechanical properties at low RFP concentrations [26].

The loss factor (tan δ), representing the ratio of viscous to elastic responses, provides crucial insights into network stability (Figure 5C). While all samples showed tan δ values below 0.5, indicating predominantly solid-like behavior, significant variations were observed across different RFP concentrations. R0.025 exhibited the highest tan δ value despite its high storage modulus, indicating a less stable network structure, where physical and covalent crosslinks coexist without optimal organization. Conversely, R0.1 showed the lowest tan δ value, reflecting the formation of a more stable network structure dominated by uniform covalent crosslinks. Lower tan δ values indicate more elastic behavior, which is characteristic of a more stable network structure [27].

Temperature-dependent measurements revealed particularly valuable information about network stability and the transition from physical to chemical crosslinking (Figure 5D). R0.025 demonstrated the most pronounced decrease in the storage modulus with increasing temperature, consistent with the description of the temperature-dependent behavior in physical networks [26]. This significant temperature sensitivity suggests a network primarily stabilized by physical interactions that were easily disrupted by thermal energy. In contrast, R0.05 and R0.075 maintained higher storage modulus retention at elevated temperatures, indicating the presence of strong covalent bonds that provided thermal stability. This thermal behavior provides strong evidence for the transition from a physically dominated to a covalently dominated network structure with increasing RFP concentration, aligning with established theories on the thermal stability differences between physical and chemical networks [26].

This rheological behavior further explained the non-linear trends observed in tensile testing results (Figure 4), particularly the fluctuations in Young’s modulus with increasing RFP concentration. The transitional network behavior at the 0.075% RFP concentration seen in the mechanical testing corresponded with the evolving viscoelastic properties demonstrated in these rheological measurements.

The comprehensive rheological analysis under small deformation conditions thus complemented and explained the large deformation tensile testing results, providing a more complete understanding of the network evolution with increasing RFP concentration. The apparent contradictions between the small and large deformation behaviors are resolved when considering the fundamental physics of polymer networks under different deformation regimes, highlighting the complexity of network structure development in photocrosslinked systems.

### 3.4. Interfacial Adhesion Mechanisms and Performance

**Figure 6:**
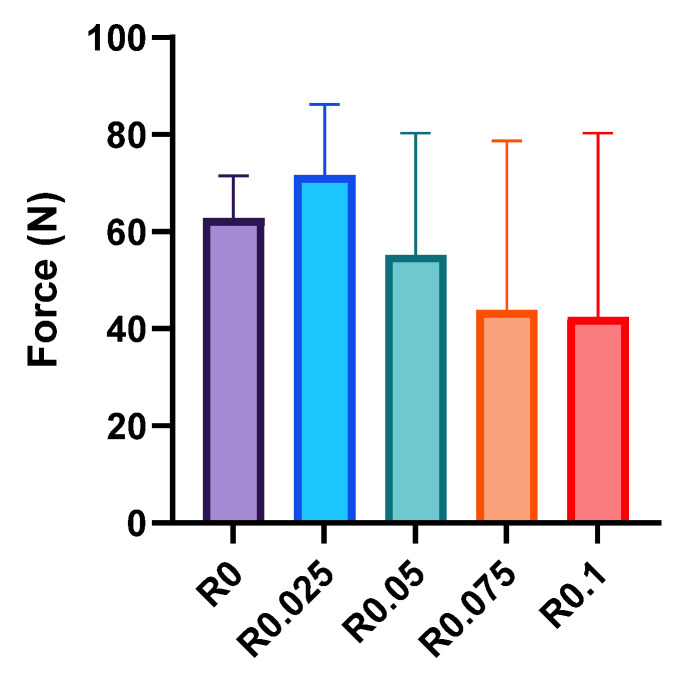
Lap shear adhesion strengths of polyurethane films with various RFP concentrations (R0–R0.1) measured using PET substrates (n = 3).

The adhesion properties of photocrosslinked films were evaluated through systematic lap shear testing using PET substrates (Figure 6). The control film (R0) exhibited a moderate adhesion strength of 62.8 ± 7.1 N, which increased slightly to 71.6 ± 11.9 N at a low RFP concentration (R0.025). However, as the RFP concentration increased further, the adhesion strength gradually decreased to 55.2 ± 20.5 N, 43.9 ± 28.5 N, and 42.4 ± 30.9 N for R0.05, R0.075, and R0.1, respectively.

This trend in adhesion behavior can be understood through the evolution of the network structure and molecular mobility at the interface. At a low RFP concentration (R0.025), the network retained significant physical interactions while incorporating a limited number of covalent bonds. Such a network structure allows for sufficient chain mobility at the interface, enabling effective molecular contact and entanglement with the substrate surface [27]. This molecular-level flexibility promotes stronger adhesion through multiple types of interfacial interactions.

However, as the RFP concentration increases and the network transitions from physically dominated to covalently dominated structure, the restricted chain mobility begins to limit interfacial interactions. This behavior aligned with our rheological observations: while higher RFP concentrations created more stable networks (as evidenced by lower tan δ values), the reduced molecular mobility (shown by the temperature-dependent behavior) impaired the ability of polymer chains to achieve intimate contact with the substrate surface. This trade-off between network stability and interfacial mobility explains the observed decrease in the adhesion strength at higher RFP concentrations.

The relationship between the adhesion behavior and network characteristics revealed by the mechanical and rheological analyses provides deeper insights into the structure–property relationships of our system. The higher storage modulus and loss modulus observed in the rheological measurements for films with R0.025 correlated with their enhanced adhesion performances under small deformation conditions. This suggests that the optimal balance between the physical and covalent crosslinks at low RFP concentrations created a network structure capable of both maintaining cohesive strength and facilitating interfacial interactions.

These findings demonstrate the critical role of molecular mobility and network architecture in determining the adhesion properties. While higher RFP concentrations enhance the bulk mechanical properties through the formation of stable covalent networks, the optimal adhesion performance requires sufficient chain mobility for effective interfacial interactions. This understanding suggests that future optimization strategies should focus on maintaining adequate molecular mobility at the interface while achieving the desired bulk properties through controlled network formation.

### 3.5. Biocompatibility Assessment and Commercial Viability

**Figure 7:**
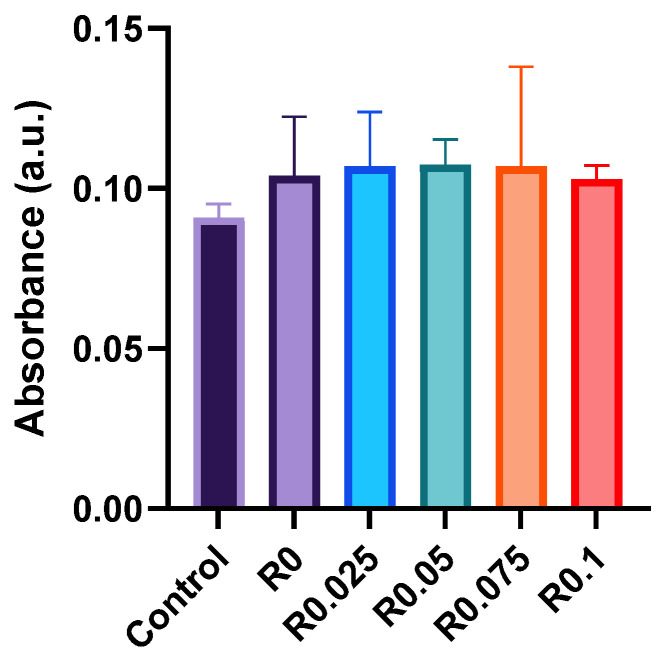
Cell viability of NIH/3T3 fibroblasts exposed to polyurethane film extracts (R0–R0.1) for 24 h, as assessed by MTT assay (n = 3).

The biocompatibility of photocrosslinked films was evaluated using NIH/3T3 fibroblast cells to assess their safety for cosmetic applications (Figure 7). Cell viability tests showed that extracts from films with various RFP concentrations (0–0.1%) maintained comparable cell viability with the control group after 24 h of exposure, although the differences were not statistically significant. The absence of cytotoxicity across all the RFP concentrations indicates the biological safety of our formulation approach.

These favorable biocompatibility results, combined with the advantages demonstrated in previous sections, position our system as a promising candidate for next-generation water-based nail polish formulations. Several key features make this technology particularly suitable for immediate commercial implementation: (1) the use of commercially available water-based polyurethanes as base materials; (2) the incorporation of RFP, an FDA-approved photoinitiator [28,29]; (3) a visible-light-mediated curing process that eliminates UV exposure risks; and (4) enhanced mechanical durability while maintaining easy peel-off functionality.

Furthermore, our formulation addresses growing market demands for environmentally conscious and user-friendly cosmetic products. The water-based nature of the system reduces volatile organic compound emissions, while the visible light curing process provides a safer alternative to traditional UV curing methods. The straightforward preparation process and compatibility with existing manufacturing protocols suggest that this technology could be readily integrated into current production lines, offering a practical pathway for developing improved nail polish products.

## 4. Conclusions

In this study, we successfully developed a visible-light-curable water-based nail polish system by strategically combining commercial water-based polyurethanes with RFP-mediated photocrosslinking. Our systematic investigation revealed a complex evolution of the network structure with increasing RFP concentration, transitioning from a physically dominated to a covalently dominated network. This transition manifested differently under various testing conditions: while the tensile testing under a large deformation showed progressive enhancement with the RFP concentration (54% increase in tensile strength and 94% increase in Young’s modulus at 0.1% RFP), the rheological analysis under a small deformation revealed more complex behavior that reflected the interplay between physical and covalent networks. The formation of a more stable network structure at higher RFP concentrations was evidenced by lower tan δ values and enhanced thermal stability, although this improved network stability led to decreased chain mobility and consequently reduced adhesion strength. This study provides fundamental insights into the structure–property relationships in photocrosslinked systems, demonstrating how the balance between physical and covalent interactions determines the material performance across different deformation regimes. The biocompatibility of all formulations and the straightforward preparation process using commercially available materials suggest the immediate potential for industrial implementation, offering a practical pathway toward environmentally conscious, user-friendly nail polish products that balance performance with safety considerations.

## Data Availability

Data will be made available on request.

## Supplementary Materials

The following supporting information can be downloaded from https://www.mdpi.com/article/10.3390/polym17060766/s1. Figure S1. Mechanical properties of polyurethane films with varying ratios. S and A represent SUD960 and Akuarane5015, respectively.

## Abbreviations

The following abbreviations are used in this manuscript:

DMEM	Dulbecco’s Modified Eagle Medium
FBS	Fetal bovine serum
FT-IR	Fourier-transform infrared
PET	Polyethylene terephthalate
RFP	Riboflavin phosphate
UTM	Universal testing machine
UV	Ultraviolet
VOC	Volatile organic compound

## References

[1] Scheers C., Andre J., Richert B. (2024). Nail cosmetology. Hand Surg. Rehabil..

[2] Wang E., Lipner S.R. (2024). Adverse Effects of Do-It-Yourself Nail Cosmetics: A Literature Review. Ski. Appendage Disord..

[3] Heaton T., Hurst L.K., Amiri A., Lungu C.T., Oh J. (2019). Laboratory Estimation of Occupational Exposures to Volatile Organic Compounds During Nail Polish Application. Workplace Health Saf..

[4] Keretetse G., Nelson G., Brouwer D. (2023). Exposure of formal and informal nail technicians to organic solvents found in nail products. Front. Public Health.

[5] Słabicka-Jakubczyk A., Lewandowski M., Pastuszak P., Barańska-Rybak W., Górska-Ponikowska M. (2023). Influence of UV nail lamps radiation on human keratinocytes viability. Sci. Rep..

[6] Mendelsohn E., Hagopian A., Hoffman K., Butt C.M., Lorenzo A., Congleton J., Webster T.F., Stapleton H.M. (2016). Nail polish as a source of exposure to triphenyl phosphate. Environ. Int..

[7] Dea M., Shanti M., Eric M., Bednar E.D., Mohannad A.-H. (2024). A systematic review of the risk of cutaneous malignancy associated with ultraviolet nail lamps: What is the price of beauty?. Eur. J. Dermatol..

[8] Maeda K., Iwashita N. (2022). Experimental Study of the Reduction in Ceramide Content in Fingernails Due to Nail Polish Remover Use. Cosmetics.

[9] Kim K.-M., Park H.-W., Shim G.-S., Jang S.-W., Kim H.-J., Chae G.-S., Shin S., Lee J.-H. (2020). Mechanical properties and decomposition performance of peelable coating containing UiO-66 catalyst and waterborne silane-terminated polyurethane dispersions. J. Mater. Sci..

[10] Peng X., Liu Y., Xin B., Guo H., Yu Y. (2020). Preparation and characterization of waterborne polyurethane nail enamel modified by silane coupling agent. J. Coat. Technol. Res..

[11] Honarkar H. (2018). Waterborne polyurethanes: A review. J. Dispers. Sci. Technol..

[12] Madbouly S.A. (2021). Waterborne Polyurethane Dispersions and Thin Films: Biodegradation and Antimicrobial Behaviors. Molecules.

[13] Dsouza R.F., Parthiban A. (2024). Crosslinking through acyl hydrazone formation by reacting water soluble polyurethanes derived from ketone diol comonomers and those containing hydrazide pendant groups. Prog. Org. Coat..

[14] Mumtaz F., Zuber M., Zia K.M., Jamil T., Hussain R. (2013). Synthesis and properties of aqueous polyurethane dispersions: Influence of molecular weight of polyethylene glycol. Korean J. Chem. Eng..

[15] Valdes B.S.G., Serro A.P., Marto J., Galhano dos Santos R., Cutrín Gómez E., Otero-Espinar F.J., Moura Bordado J., Margarida Ribeiro H. (2018). Polyurethanes as New Excipients in Nail Therapeutics. Pharmaceutics.

[16] Lee Y.B., Lim S., Lee Y., Park C.H., Lee H.J. (2023). Green Chemistry for Crosslinking Biopolymers: Recent Advances in Riboflavin-Mediated Photochemistry. Materials.

[17] Kim S.Y., Kim J.H., Kang Y., Yoo J.W., Choi J., Lee H.J. (2022). Green chemistry method for hair strengthening and setting using visible light-mediated protein crosslinking. J. Clean. Prod..

[18] Kim J.H., Kim S.Y., Choi J., Lee H.J. (2023). Visible Light-Mediated Environmentally Friendly and Universally Applicable Green Chemistry for Hair Cross-Linking. ACS Sustain. Chem. Eng..

[19] Hong M.J., Lee Y., Kyung S.J., Choi J., Lee H.J. (2024). Sustainable and durable color cosmetics: Riboflavin phosphate-mediated photo-crosslinked casein films with tannic acid. Biomater. Sci..

[20] Na K.-S., Kim D., Kim H., Koh W.-G., Lee H.J. (2024). The combined effect of epidermal growth factor and keratinocyte growth factor delivered by hyaluronic acid hydrogel on corneal wound healing. Int. J. Biol. Macromol..

[21] Lim S., Kim J.A., Chun Y.H., Lee H.J. (2023). Hyaluronic acid hydrogel for controlled release of heterobifunctional photocleavable linker-modified epidermal growth factor in wound healing. Int. J. Biol. Macromol..

[22] Lee Y., Lim S., Kim J.A., Chun Y.H., Lee H.J. (2023). Development of Thiol–Ene Reaction-Based HA Hydrogel with Sustained Release of EGF for Enhanced Skin Wound Healing. Biomacromolecules.

[23] Duval A., Avérous L. (2023). From thermoplastic polyurethane to covalent adaptable network via reversible photo-crosslinking of a biobased chain extender synthesized from caffeic acid. Polym. Chem..

[24] Zhang C., Li Y., Wu Y., Wang C., Liang J., Xu Z., Zhao P., Wang J. (2024). Key Role of Cross-Linking Homogeneity in Polyurethane Mechanical Properties: Insights from Molecular Dynamics. J. Phys. Chem. B.

[25] Rubinstein M., Colby R.H., Rubinstein M., Colby R.H. (2003). Ideal chains. Polymer Physics.

[26] Rubinstein M., Colby R.H., Rubinstein M., Colby R.H. (2003). Networks and gels. Polymer Physics.

[27] Rubinstein M., Colby R.H., Rubinstein M., Colby R.H. (2003). Entangled polymer dynamics. Polymer Physics.

[28] Spoerl E., Mrochen M., Sliney D., Trokel S., Seiler T. (2007). Safety of UVA-Riboflavin Cross-Linking of the Cornea. Cornea.

[29] U.S. Food and Drug Administration (1978). Select Committee on GRAS Substances (SCOGS) Opinion: Riboflavin, Riboflavin-5′-Phosphate.

