# Supplementary material for: Enhanced Mechanical Stability of Water-Based Peel-Off Nail Polish Through Riboflavin Phosphate-Mediated Visible Light Photocrosslinking
Source: Polymers (Basel). 2025 Mar 14;17(6):766. doi: 10.3390/polym17060766 (PMC11946657; doi:10.3390/polym17060766)
Supplement: Supplementary file 1 [file polymers-17-00766-s001.zip › polymers-3498576-supplementary.pdf]

polymers

MDPI

Supplementary Materials

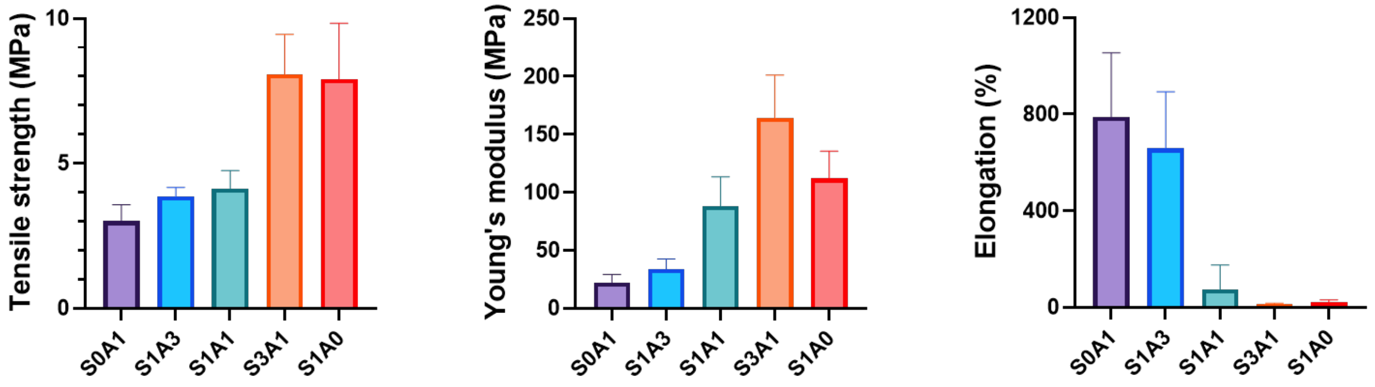

Figure S1. Mechanical properties of polyurethane films with varying ratios. S and A represent SUD960 and Akuarane5015, respectively.
